# Efficient removal of crystal violet using Fe_3_O_4_-coated biochar: the role of the Fe_3_O_4_ nanoparticles and modeling study their adsorption behavior

**DOI:** 10.1038/srep12638

**Published:** 2015-07-29

**Authors:** Pengfei Sun, Cai Hui, Rashid Azim Khan, Jingting Du, Qichun Zhang, Yu-Hua Zhao

**Affiliations:** 1 College of Life Sciences, Zhejiang University, 310058 Hangzhou, Zhejiang, PR China; 2 School of Forestry and Biotechnology, Zhejiang Agriculture and Forestry University, 311300 Lin’an, Zhejiang, PR China; 3 College of Environmental and Resource Sciences, Zhejiang University, 310058 Hangzhou, Zhejiang, PR China

## Abstract

Biochar shows great promise for use in adsorbing pollutants. However, a process for enhancing its adsorption capacity and re-collection efficiency is yet to be further developed. Hence, in this study, we developed a type of biochar coated with magnetic Fe_3_O_4_ nanoparticles (i.e., magnetic biochar (MBC)) and assessed its use for crystal violet (CV) adsorption as well as its recycling potential. The coating of Fe_3_O_4_ nanoparticles, which was not only on the surface, but also in the interior of biochar, performed two functions. Firstly, it produced a saturation magnetization of 61.48 emu/g, which enabled the biochar being efficiently re-collected using a magnet. Secondly, it significantly enhanced the adsorption capacity of the biochar (from 80.36 to 99.19 mg/g). The adsorption capacity of the MBC was determined to be the largest by so far (349.40 mg/g) for an initial CV concentration of 400 mg/L, pH of 6.0, and temperature of 40 °C, and the adsorption capacity of re-collected MBC was 73.31 mg/g. The adsorption of CV by the MBC was found to be a spontaneous and endothermic physical process in which the intraparticle diffusion was the limiting step. These findings inspire us to use other similar materials to tackle the menace of pollutions.

Synthetic dyes are widely used in the textile, paper, printing, food, and other industries, and vast amounts are released into the environment together with industrial wastewater^1^. Crystal violet (CV), which is a type of synthetic dye with complex organic structures, is widely used in the textile industry and often present in industrial wastewater. However, its undesirable and illegal release into the environment poses a significant threat to human health because organic dyes are teratogenic, carcinogenic, and mutagenic^2^. The development of a process for the efficient treatment of CV-polluted wastewater is therefore of utmost importance.

The adsorption technique is currently widely used to remove contaminants from industrial wastewater owing to its many advantages, which include simplicity, high efficiency, and ease of use^3^,^4^. Moreover, some types of biochars that can be easily produced from agricultural remains such as cornstalk, corncob, and rapeseed oil cake, have been shown to be efficient adsorbents for the removal of dyes^5^,^6^, chromium^7^, heavy metals^8^, and pentachlorophenol^9^.

However, as with other powdered adsorbents, it is difficult to separate powdered biochar from an aqueous solution for reuse. In addition, the original adsorption capacity of biochar is quite low and enhancement is thus required. The introduction of magnetic nanoparticles into the adsorbent by different methods has been proposed to solve these problems^10^,^11^,^12^.

The current study was aimed at producing a novel MBC adsorbent and using it to treat CV-polluted wastewater. The characteristics of the MBC were analyzed by Fourier transform infrared spectroscopy, scanning and transmission electron microscopy, elemental analysis, X-ray diffraction analysis, and magnetic hysteresis loop analysis. The adsorption properties of the MBC to CV, including the adsorption kinetics and the isothermal and thermodynamic properties, were also investigated.

## Results and Discussion

### Characterization of MBC

**Figure 1:**
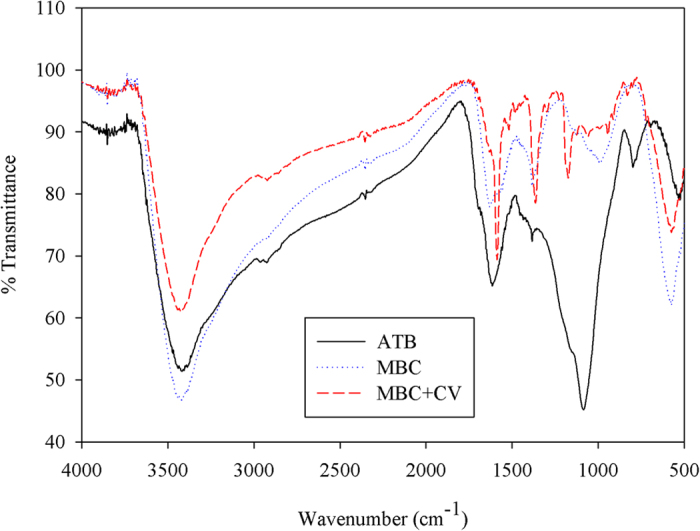
Fourier transform infrared spectra of acid-treated biochar (ATB), Fe_3_O_4_ coated biochar (MBC), and crystal violet-loaded Fe_3_O_4_ coated biochar (MBC + CV).

The FTIR spectra of the acid-treated biochar (ATB), virgin MBC and CV-loaded MBC are shown in Fig. 1. The main bands that represent the vibration of the functional groups in the ATB are as follows: -OH: 3416 cm^−1^, aromatic C = C and C = O: 1615 cm^−1^, and C-O-C: 1085 cm^−1^. Compared to previously reported main bands of biochars^13^,^14^, the functional groups in the ATB are relatively few. This may be because of the loss of some organic residues in our biochar during the acid treatment. Comparison of the spectra of the ATB and MBC reveals that the strong absorption peak at 1085 cm^−1^ in the former is replaced by three new absorption peaks at 1383, 985, and 572 cm^−1^ in the latter. The peak only exists in the spectra of the MBC at 572 cm^−1^, and is caused by the Fe-O of iron oxide. This indirectly confirmed the presence of Fe_3_O_4_ in the MBC^5^. The low-intensity peak at 1383 cm^−1^ reflects the presence of the hydroxyl groups attached to the benzene ring and the deformation of the -CH_3_ group^15^. The peak at 985 cm^−1^ in the spectrum of the MBC reflects the stretching vibration of the C-C in ethane. The adsorption of CV onto MBC caused some changes in some bands of the CV-loaded MBC spectrum (mainly within 1800–800 cm^−1^) relative to that of the virgin MBC. The peak at 572 cm^−1^ in the spectrum of the CV-loaded MBC indicates that the Fe-O of iron oxide is still present, and the representative peak of -NH at 1587 cm^−1^ confirms the adsorption of CV onto the MBC. Overall, the differences observed among the FTIR spectra of the ATB, MBC, and CV-loaded MBC are indicative of their different functional groups. For example, C-O-C could be responsible for the loading of Fe_3_O_4_ onto ATB to produce MBC, and functional groups such as the aromatic C = C and C = O, -OH, -CH_3_, and -CH = CH_2_ could be responsible for the adsorption of CV onto MBC.

**Figure 2:**
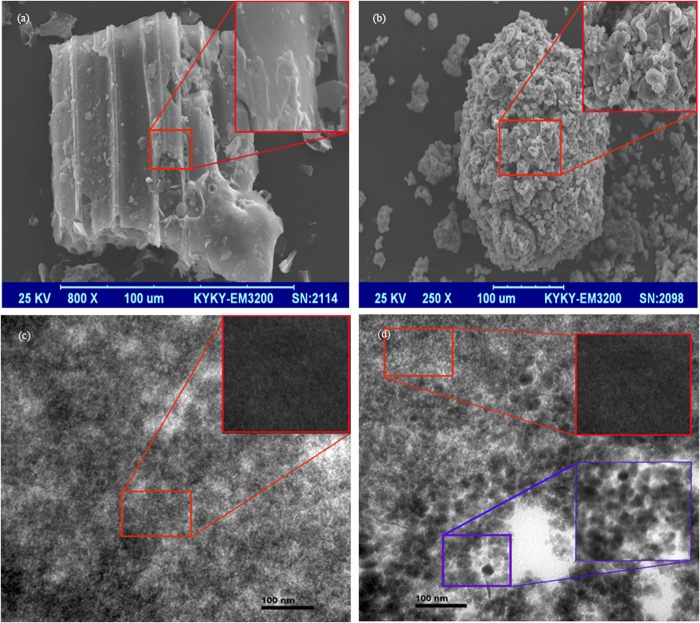
Scanning electron microscope (SEM) and transmission electron microscope (TEM) images of two different biochars. (**a**) SEM image of acid-treated biochar (control), (**b**) SEM image of Fe_3_O_4_ coated biochar, (**c**) TEM image of acid-treated biochar, (**d**) TEM image of Fe_3_O_4_ coated biochar. Phases of ATB and MBC highlighted in small boxes are magnified 1000 times in big boxes.

TEM, SEM, magnetic hysteresis loop analysis, XRD, and elemental analysis were used to further confirm the existence of magnetic iron oxide (Fe_3_O_4_) in the MBC. The SEM and TEM results are shown in Fig. 2. Figure 2a shows the SEM image of the ATB, the smooth surface of which can be observed in the magnified image. As can be seen from the SEM image in Fig. 2b, the surface of the MBC was entirely different from that of the ATB. As can be observed from the magnified image, the aggregation of many partial substances on the surface of the MBC made it rugged. We concluded that granulated iron oxide existed on the surface of the ATB. Figure 2c,d respectively show the TEM images of the ATB and MBC. The magnified image in Fig. 2c reveals that the interior of the ATB comprised only one phase, although this was changed when the ATB was converted into MBC. The TEM image of the MBC in Fig. 2d reveals the existence of two inner components, namely, Fe_3_O_4_ nanoparticles (see the magnified image in the blue box in Fig. 2d) and biochar (see magnified image in red box in Fig. 2d). We thus concluded that Fe_3_O_4_ nanoparticles not only existed on the surface of the biochar, but also in its interior. These results indicate that the novel method for coating biochar with Fe_3_O_4_ nanoparticles is highly effective.

**Figure 3:**
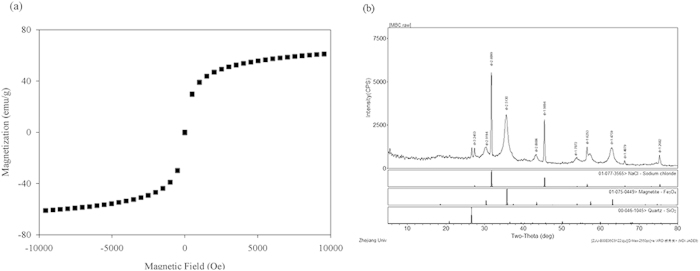
Magnetism and structural analysis of Fe_3_O_4_ coated biochar. (**a**) Magnetic hysteresis loop obtained at room temperature, (**b**) X-ray diffraction pattern.

To investigate the magnetic properties of the MBC, its magnetic hysteresis curve was obtained at room temperature. The sample curve in Fig. 3a confirms the ferromagnetic property, and indicates a saturation magnetization of 61.48 emu/g, which is higher than those of Fe_3_O_4_ nanoparticles (9.9 emu/g) and Fe_3_O_4_-coated cells (14.3 emu/g)^16^,^17^. In addition, the magnetic separation properties of the MBC and ATB were observed for a total of 3 days. It was found that the entire MBC was easily and stably re-collected by a magnet, whereas the ATB remained dispersed in the solution and cannot be re-collected by a magnet (see Fig. S1 in the supplementary information section). This confirmed the feasibility of using magnetic separation for the re-collection of powdered MBC after its initial use.

The XRD pattern confirms that MBC consists of amorphous and crystalline phases. The amorphous phase is supposed to be C, and the crystalline phase consists of Fe_3_O_4_, NaCl, and SiO_2_ (Fig. 3b), which verified the coating of Fe_3_O_4_ nanoparticles in biochar. The quantitative elemental compositions of the ATB and MBC are listed in Table 1. The lower C, H, and N contents of the MBC (especially the C content) compared with the control sample (ATB) are due to the introduction of Fe_3_O_4_ into the ATB. These overall results further prove the existence of Fe_3_O_4_ in the MBC.

The average pore radiuses of the ATB and MBC were measured and found to be 5.77 and 9.85 nm. This indicated that the introduction of the Fe_3_O_4_ nanoparticles increased the average pore radius of the biochar. These results are consistent with the findings of Chen *et al.*^5^. Additionally, the measurement of the zeta potentials revealed that the introduction of the Fe_3_O_4_ nanoparticles reduced the zeta potential of the biochar. The zeta potentials of the ATB and MBC were determined to be −22.7 ± 1.31 and −41.07 ± 3.23 mV.

### Adsorption capacities of original biochar (OB), ATB and MBC

**Figure 4:**
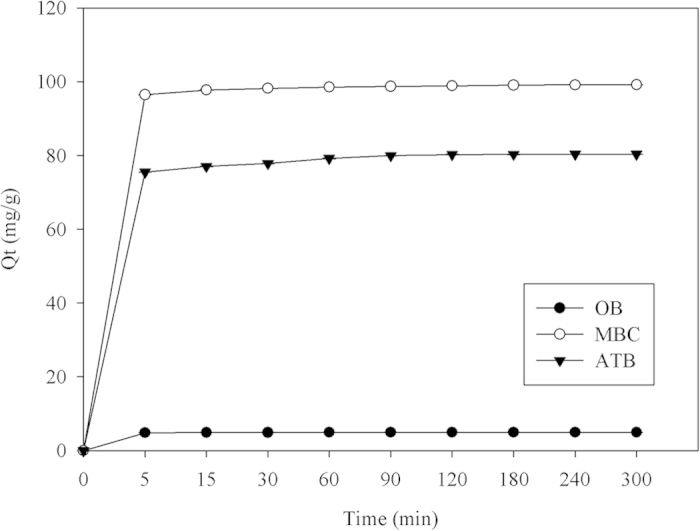
Comparison of adsorption capacities of original biochar (OB), acid-treated biochar (ATB), and Fe_3_O_4_ coated biochar (MBC).

The adsorption capacities of the OB, ATB, and MBC were determined as shown in Fig. 4. The CV adsorption processes of the OB, ATB, and MBC all rapidly attained equilibrium (within 5 min). However, the maximal adsorption capacities of these three adsorbents for an initial CV concentration of 100 mg/L and pH of 6.0 were remarkably different, being 4.95, 80.36, and 99.38 mg/g, respectively. In our experiment, OB was first pretreated in concentrated hydrochloric acid to produce ATB, which was then used to prepare MBC. Comparison of the adsorption capacities revealed that pretreatment using concentrated hydrochloric acid significantly contributed to the enhancement of the adsorption capacity of OB. We speculated that more adsorption sites on biochar for CV were released after the concentrated hydrochloric acid treatment. Furthermore, we interestingly found that the coating of magnetic Fe_3_O_4_ nanoparticles on the ATB maximized the adsorption capacity, almost resulting in saturation. Based on these above results, we conclude that the coating of Fe_3_O_4_ nanoparticles performed two functions. Firstly, it produced a saturation magnetization of 61.48 emu/g, which enabled the biochar being efficiently re-collected using a magnet. Secondly, it significantly enhanced the adsorption capacity of the biochar (from 80.36 to 99.19 mg/g). We speculate two reasons for the enhancement of the adsorption capacity of the biochar by the Fe_3_O_4_ nanoparticles. The first is that the introduction of the nanoparticles increased the average pore radius of the biochar^5^, and the second is that the introduction of the nanoparticles decreased the zeta potential. The latter probably enhanced the electrostatic interaction between the negatively charged MBC and the cationic CV, thereby improving the adsorption capacity of the biochar.

### Effect of pH on adsorption capacity

**Figure 5:**
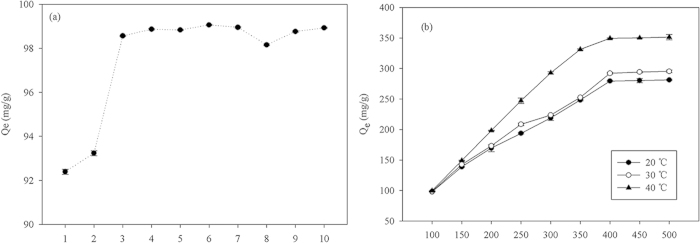
Effects of (**a**) initial pH and initial dye concentration, and (**b**) temperature, on crystal violet adsorption capacity of Fe_3_O_4_ coated biochar.

As can be seen from Fig. 5a, the pH of the CV solution did not substantially affect the adsorption capacity of the MBC. With the exception of the cases of pH values of 1 and 2, there are no significant differences among the adsorption capacities of the MBC under the different pH conditions. For the same speed of 200 rpm and temperature of 40 °C, the *Q*_*e*_ for the different pH values of 1.0 to 10.0 were 92.38, 93.24, 98.53, 98.84, 98.81, 99.13, 98.92, 98.13, 98.78, and 98.89 mg/g, respectively (Fig. 5a). Apparently, a pH of 6.0 is optimal for CV adsorption by MBC owing to its affording the highest adsorption capacity. Cationic dyes including CV are favorably adsorbed onto negatively charged adsorbents^18^. The high concentration of the H^+^ ions and the small ionic radius cause the ions to effectively compete with the CV molecules, and this enables easier adsorption of the H^+^ ions by the MBC compared to the CV, resulting in reduced adsorption of the latter at low pH values (e.g., pH = 1 or 2). Moreover, the repulsion between the protonated adsorbent (adsorbents are usually protonated in strongly acidic solutions) and the CV molecules dominate the adsorption process, thereby reducing the adsorption capacity^1^,^14^. This explains the pH of 6.0 being optimal for CV adsorption by MBC.

### Effect of temperatures and CV concentrations on adsorption capacities

Figure 5b shows the effects of temperatures and CV concentration on the adsorption process. As can be seen, the CV adsorption capacity of the MBC increased rapidly with increasing CV concentration from the initial value of 100 to 400 mg/L, after which it became constant. As Fig. 5b also reveals, the temperature remarkably affected the CV absorption. For initial CV concentrations of 100–500 mg/L, the adsorption capacity increased with increasing temperature from 20 to 40 °C, with a temperature of 40 °C being optimal. It can also be deduced that the CV adsorption by MBC is an endothermic process. It is speculated that the increased temperature decreased the viscosity of the CV solution and thus increased the adsorption capacity^19^. Compared to previously reported CV adsorption capacities (see Table 2), the adsorption capacity of the MBC for CV (349.40 mg/g) is higher, and is actually almost 100% higher than the highest previously reported adsorption capacity (182.15 mg/L)^1^. This indicates that the MBC is extremely efficient in adsorbing CV.

### Adsorption kinetics modeling studies

**Figure 6:**
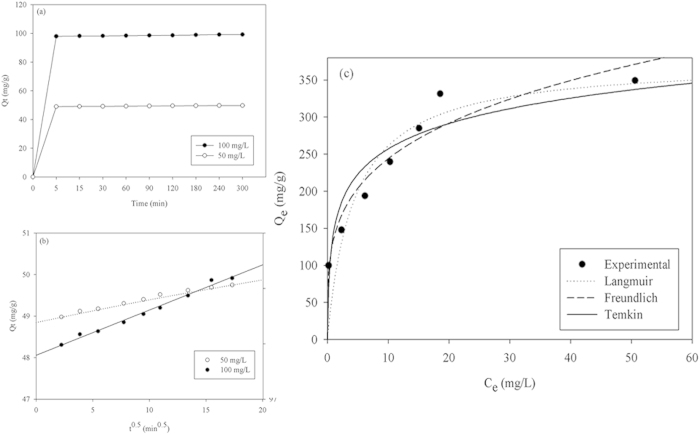
Adsorption kinetics (**a**) Effect of contact time on adsorption of crystal violet onto Fe_3_O_4_ coated biochar for different initial dye concentrations (50 and 100 mg/L), (**b**) Weber-Morris plots of adsorption of crystal violet onto Fe_3_O_4_ coated biochar for initial dye concentrations of 50 and 100 mg/L, and (**c**) Isotherms for adsorption of crystal violet onto magnetic biochar (25 mg adsorbent, pH 6, 40 °C, 4 h).

CV adsorption onto MBC as a function of time was investigated using two different initial concentrations of 50 and 100 mg/L. As shown in Fig. 6a, the adsorption rates of CV for the two initial concentrations rapidly attained equilibrium within 5 min. Because the determined coefficients (R^2^) of the pseudo-first-order (P-F-O) Lagergen were less than 0.50, P-F-O could not be used to describe the adsorption kinetics. The pseudo-second-order (P-S-O) model was therefore used to investigate the kinetics of the adsorption of CV by MBC, and the Weber-Morris intraparticle diffusion model was used to analyze the kinetics results to determine the adsorption mechanism. The equation of the P-S-O kinetics model is as follows:
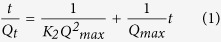
where *K*_*2*_ is the equilibrium rate constant of the P-S-O adsorption (g/mg·min), *Q*_*max*_ is the maximum adsorption capacity (mg/g) for the P-S-O adsorption, and *Q*_*t*_ is the adsorption capacity (mg/g) at any adsorption time *t* (min). Based on the R^2^ values in Table 3 (R^2^ > 0.98 for both tested conditions), the P-S-O model satisfactorily fits the process of CV adsorption onto MBC. The *Q*_*max*_ values estimated by the P-S-O kinetics model (*Q*_*eq,cal*_ in Table 3) for the two initial dye concentrations were 49.11 and 98.11 mg/g, respectively, which agree well with the experimentally determined values (*Q*_*eq,exp*_ in Table 3) of 49.75 and 99.18 mg/g, respectively. These results suggest that the boundary layer resistance is not a rate-limiting factor because the CV adsorption process is in accordance with the P-S-O kinetics model^19^.

The Weber-Morris model equation is as follows:
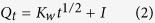
where *Q*_*t*_ is the adsorption capacity (mg/g) at adsorption time *t* (min), *K*_*w*_ is the intraparticle rate constant, *t*^*1/2*^ is the square root of the time, and *I* is the intercept.

The Weber-Morris model could be used to investigate the mass transfer mechanism in the CV-MBC system. Figure 6b shows the Weber-Morris plots, the parameters of which are summarized in Table 4. The Weber-Morris plots of the adsorption for CV concentrations of 50 and 100 mg/L are both linear and do not pass through the origin. This bears out the significance of intraparticle diffusion that occurs during the adsorption of CV onto MBC. The intercept gives an idea of the thickness of the boundary layer; the larger the intercept, the thicker the boundary layer and its effect^20^. The large intercepts in the present results for initial CV concentrations of 50 and 100 mg/L (Table 4) indicate that the effect of boundary layer on the adsorption process cannot be ignored. In addition, the intercept for a concentration of 100 mg/L is higher than that for a concentration of 50 mg/L (Table 4), indicating that the boundary layer effect on the adsorption process is greater for a higher concentration of the dye. In general, the adsorption process in the present study may have involved the diffusion of CV molecules from the external layer to the surface of the MBC, the internal diffusion of the CV molecules within the pores, and the adsorption of CV to available sites on the interior MBC surfaces^21^. On saturation of the adsorption sites, the adsorption rate during the final stages of the process begins to decrease owing to the attainment of equilibrium.

### Adsorption isotherm modeling studies

To examine the isotherm behaviors of CV adsorption onto the MBC, the theories of Langmuir, Freundlich, and Temkin were employed. The equations of the theories are as follows:
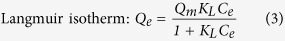

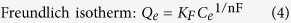

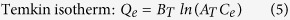


where *C*_*e*_ is the CV concentration at equilibrium (mg/L), *Q*_*e*_ is the adsorption capacity at equilibrium (mg/g), *Q*_*m*_ is the maximum adsorption capacity (mg/g), *K*_*L*_ is the Langmuir constant (L/mg), *K*_*F*_ is the Freundlich adsorbent capacity, *n*_*F*_ is the heterogeneity factor, *A*_*T*_ is the Temkin equilibrium binding constant (L/mol), and *B*_*T*_ is the Temkin constant.

The isotherm information obtained by using these three models to analyze the data are shown in Fig. 6c and summarized in Table 5. The Langmuir theory assumes homogeneous adsorption, which implies that when a dye molecule occupies an adsorption site, no further adsorption can occur at that site^22^. The Langmuir model used for this study has high R^2^ values (Table 5), which indicate homogeneous adsorption of CV onto the surface of the MBC. The attainment of the maximum values of *Q*_*max*_ and *K*_*L*_ (356.71 mg/L and 0.870 L/mg, respectively) at 40 °C indicates that the molecules of CV exhibited the highest affinity for MBC at 40 °C. This is in agreement with the experimental results shown in Table 5. The favorability of the adsorption can be determined from the obtained values of the essential characteristics of the Langmuir isotherm (*R*_*L*_). *R*_*L*_ can yield an isotherm shape that is either unfavorable (*R*_*L*_ > 1), linear (*R*_*L*_ = 1), favorable (0 < *R*_*L*_ < 1), or irreversible (*R*_*L*_ = 0)^23^. The equation of *R*_*L*_ is as follows:
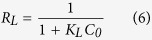
where *K*_*L*_ and *C*_*0*_ are respectively the Langmuir constants (L/mg) and the initial dye concentration (mg/L). All the *R*_*L*_ values (data not shown) calculated using equation (6) were within 0–1, indicating that the adsorption process was favorable.

In addition, the values of the Freundlich constant *n* for CV were all within 2–10 (see Table 5), which indicates favorable adsorption. The high values of *K*_*F*_ for CV in Table 5 imply that MBC has a high capacity for adsorbing the dye from a solution. These trends are similar to those observed for the adsorption of Remazol Blue reactive dye by dried yeasts^24^, and of Acid Black 172 and Congo Red by *Penicillium* YW 01^19^.

In contrast to the logarithmic relationship of the Freundlich isotherm, the Temkin isotherm assumes that the adsorption heat decreases in a linear manner. The relatively low *A*_*T*_ values for the adsorption of CV onto MBC under different temperatures indicate a low potential of MBC for the adsorption of CV. This may be attributed to the low electrostatic attraction between CV and MBC at different temperatures^25^. The values of the Temkin constant, which is related to the adsorption heat of CV at given different temperatures, increased with increasing adsorption temperature. The obtained values of *B*_*T*_ (*B*_*T*_ < 8.0 kJ/mol, in Table 5) indicate that the interaction between CV and MBC was a weak physisorption. This is because the typical range of the bonding energy for an ion-exchange mechanism (chemisorption) is 8–16 kJ/mol^14^.

### Thermodynamic properties modeling studies

The thermodynamic properties were investigated to determine whether the adsorption process occurred spontaneously. The thermodynamic parameters, namely, the Gibbs free change (*ΔG*^*0*^), enthalpy change (*ΔH*^*0*^), and entropy change (*ΔS*^*0*^), were calculated using the following equations:
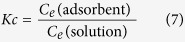

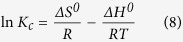

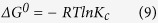
where *R* (=8.314 J/mol·K) is the ideal gas constant, *M* (=407.98 g/mol) is the molar mass of CV, *T* (K) is the absolute temperature, *K*_*c*_ is the equilibrium constant of adsorption, *C*_*e*_ (adsorbent) is the concentration of the adsorbed CV on the adsorbent at equilibrium, and *C*_*e*_ (solution) is the concentration of the CV solution at equilibrium. The values of *ΔH*^*0*^ and *ΔS*^*0*^ were determined from the slopes (−ΔH^0^*/R*) and intercepts (*ΔS*^*0*^*/R*) of the plots of ln *Kc* versus 1/T.

The values of these thermodynamic parameters were determined at three different temperatures and the results are given in Table 6. The values of these parameters are an indication of the type of adsorption force based on the following general guidelines: 4 < *ΔH*^*0*^ < 10 kJ/mol: van der Waals force, 2 < *ΔH*^*0*^ < 40 kJ/mol: hydrogen-bonding force, 2 < *ΔH*^*0*^ < 29 kJ/mol: dipole−dipole interaction, *ΔH*^*0*^ > 60 kJ/mol: chemical bonding force, *ΔH*^*0*^ ≈ 40 kJ/mol: exchange of dentate, −20 < *ΔG*^*0*^ < 0 kJ/mol: physical adsorption, and −400 < *ΔG*^*0*^ < −80 kJ/mol: chemical adsorption^1^,^14^. As indicated in Table 6, the *ΔG*^*0*^ values were negative for all three considered temperatures, and were within the range of −20 < ΔG^0^ < 0 kJ/mol, indicating that the adsorption process was a spontaneous physical process. As can be observed, the *ΔG*^*0*^ values decrease with increasing temperature, and this suggests that the degree of spontaneity of the adsorption may be low at elevated temperatures^14^. The positive value of *ΔH*^*0*^ of 42.68 kJ/mol, which is close to 40.00 J/mol, indicates that the adsorption process is induced by dentate exchange and physical interaction, and is endothermic. This is in agreement with the previously discussed results of the kinetic experiments performed at three different temperatures. Based on the above results, it is speculated that the amount of active sites available on the MBC increased with increasing temperature, resulting in enhanced adsorption capacity. The positive value of *ΔS*^*0*^ of 151.23 J/K·mol confirms that the randomness of the CV molecules increased at the solid-solution interface during the adsorption process, indicating high affinity of CV for MBC. These observations also suggest the occurrence of structural changes in the sorbent and sorbate^14^, as observed in previous studies^14^,^26^.

### Reusability of MBC

Reusability, which is an indication of economy, is an important property of an adsorbent. Desorption of the MBC was performed before its reuse, and it was observed that the process was instantaneous and equilibrium was attained within a very short time (several seconds). The desorption efficiency was 78.19%, and the adsorption capacity of the re-collected MBC after desorption treatment was determined to be 73.31 mg/L. This indicates a slight decrease in the adsorption capacity compared to that of the original MBC (99.19 mg/g). We speculate that the decrease may be due to the presence of a few sites on the MBC where the adsorption of the CV molecules could not be reversed by the absolute ethyl alcohol used for the desorption treatment^1^. Therefore, we believe that the improvement of the desorption efficiency to make available much more adsorption sites on the MBC would effectively enhance the adsorption capacity of the re-collected MBC.

## Methods and Materials

### Materials

The corn stalks collected from Jinhua, Zhejiang, China were first air dried and then cut into small pieces of 10–20 cm. The air dried material was then placed in closed perforated stain less steel boxes and heated in a muffle furnace at 400 °C for 120 min. After carbonization, the residue was ground, passed through a 0.154-mm sieve, and used to produce the OB. The OB was initially treated in concentrated hydrochloric acid at a temperature of 30 °C on a magnetic stirrer for 24 h, and the ATB was then washed in distilled water to a neutral pH before being used to prepare the magnetic biochar.

The MBC was prepared using ATB as the raw material and magnetite (Fe_3_O_4_ nanoparticles) as the objective magnetic medium. This was done by modified chemical co-precipitation at a temperature of 80 °C on a magnetic stirrer for 1 h^5^,^16^. 9.9 g of ferrous chloride and 27 g of ferric chloride were dissolved in 100 mL of deionized water under vigorous magnetic stirring at a temperature of 80 °C, and then a 5 mol/L NaOH solution was added dropwise to adjust the pH of the solution to 10.5 g of ATB was thereafter added to the mixture. The stirring was then continued for 1 h. The mixture was subsequently separated by centrifugation at 3600 *g* for 10 min and the precipitate was oven-dried at 60 °C to a constant weight. The magnetic biochar was then collected and identified as MBC.

### Preparation of dye solution and determination of dye concentration

The employed CV was obtained from Shanghai Sangon Biological Engineering Technology & Services Co., Ltd., China. A 1000 mg/L CV stock solution was prepared in deionized water and parts of the solution were then diluted to different concentrations. An optical density of 591 nm was used to measure the concentration of the CV in the solutions, and the CV calibration curve was prepared by measuring the absorbance of the CV solutions of different concentrations.

### Characterization of absorbents

The Fourier transform infrared (FTIR) spectra of the ATB, the virgin MBC, and the CV-loaded MBC were recorded in the region of 4000–400 cm^−1^ using a PerkinElmer spectrum spectrophotometer. The transmission electron microscopy (TEM) micrographs of the ATB and MBC were also obtained using a JEM-2010 (HR) electron microscope (JEOL, Japan) with an accelerating voltage of 200 kV. In addition, scanning electron microscopy (SEM) analyses of the ATB and MBC were performed using a JEOL JSM5600 LV scanning electron microscope. The magnetic hysteresis loop of the MBC was measured using a Magnetic Measurements Variable Field Translation Balance (MPMS-XL-5, America), and the saturation magnetization was obtained from the hysteresis loop produced by applying a magnetic field of between −10 and +10 kOe. The magnetic separation property of MBC after being used for the adsorption of CV was examined by means of a magnet (N35, Zhuo Yue Ci Dian, China). The power X-ray diffraction (XRD) patterns were recorded on a Rigaku D/MAX-2550PC X-ray diffractometer equipped with Cu Kα radiation (λ = 1.54059 A) over a *2θ* range of 10°–80°. Elemental (C, H, and N) analyses of the ATB and MBC were conducted using an EA112 CHN elemental analyzer (Thermo Finnigan). The zeta potentials of ATB and MBC suspensions (1 g/L, pH = 6.0) were measured by a zeta potential analyzer (Zetasizer Nano-zs90, Malvern Co., U.K.), while the average pore radiuses of ATB and MBC were determined by multipoint BET analysis of the adsorption data points with relative pressures of 0.05–0.3^5^.

### Adsorption experiments

In each experiment, 25 mg of MBC was placed in a 100 mL conical flask and mixed with 25 mL of CV solution. The adsorption capacities of OB, ATB and MBC were first determined for an initial CV concentration of 100 mg/L, pH of 6.0, and temperature of 40 °C. To measure the sorption kinetics, CV solutions with two different initial concentrations (50 and 100 mg/L) were used, and the adsorption was measured at time intervals ranging between 5 and 300 min. To determine the sorption isotherms, MBC was shaken in CV solutions of different initial concentrations (ranging between 100 and 500 mg/L) for 240 min at different temperatures (20, 30, and 40 °C). All the solutions above had a pH of 6.0. The effect of the hydrogen ion concentration on the adsorption process was also examined using different pH values ranging between 1.0 and 10.0, which were achieved by adding varying amounts of NaOH (0.5 mol/L) and HCl (0.5 mol/L). The CV concentration in this case was maintained at 100 mg/L. In all the experiments, the adsorbent was centrifuged at 10800 *g* for 2 min after the mixtures had been shaken in a controlled shaker (200 rpm) for a certain time at the specified temperatures. The CV concentrations in the supernatants were then determined by an ultraviolet spectrophotometer.

The adsorption capacity of MBC, *Q*_*e*_ (mg/g), was calculated using the following equation:
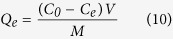
where *V* is the solution volume (mL), *M* is the mass of MBC (mg), and *C*_*0*_ and *C*_*e*_ (mg/mL) are respectively the dye concentrations at the initial time and some other specified time.

Each of the experiments was performed three times and the data were analyzed by the Sigma Plot software 10.0. The process of the adsorption of CV onto MBC was modeled by the Curve Expert software.

### Desorption and reusability of MBC

A desorption experiment was performed to investigate the reusability of the MBC. 25 mg of CV-loaded MBC (the MBC was loaded with CV by immersion in a 100 mg/L CV solution) was placed in a 100 mL conical flask and mixed with 25 mL of absolute ethyl alcohol. The process was repeated twice with the mixture agitated at 200 rpm at 30 °C for 2 h. The changes in the OD_591 nm_ of the supernatants were used to evaluate the desorption efficiency. The desorption efficiency was calculated using Eq. 11 and 12. After the desorption, the MBC was re-collected and dried in an oven at 60 °C. The adsorption capacity of the re-collected MBC was then determined by the method described above (100 mg/L CV solution, pH 6.0, 40 °C, 4 h). The adsorption capacity was calculated using Eq. 10.
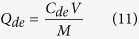
where *V* is the absolute ethyl alcohol volume (mL), *M* is the mass of the MBC (mg), and *C*_*de*_ (mg/mL) is the final dye concentrations in the absolute ethyl alcohol.
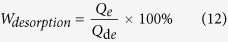
where *W*_*desorption*_ is the desorption efficiency (%), and *Q*_*e*_ and *Q*_*de*_ (mg/mL) are respectively the adsorption and desorption capacities of the MBC.

## Conclusion

In this present study, we note that the coating of biochar with magnetic Fe_3_O_4_ nanoparticles not only significantly enhanced the adsorption capacity, but also substantially increased the magnetism, thus enabling the re-collection of the biochar by a magnet. Moreover, the adsorption capacity for CV by the MBC was found to be the largest, and the adsorption was a spontaneous and endothermic physical process in which the intraparticle diffusion was the limiting step. Additionally, the re-collected MBC was shown to be reusable with a CV adsorption capacity of 73.31 mg/g. The findings of this study confirm the potential of the developed magnetic biochar for use as a sorbent in the treatment of cationic CV-polluted wastewater.

## Additional Information

**How to cite this article**: Sun, P. *et al.* Efficient removal of crystal violet using Fe_3_O_4_-coated biochar: the role of the Fe_3_O_4_ nanoparticles and modeling study their adsorption behavior. *Sci. Rep.* **5**, 12638; doi: 10.1038/srep12638 (2015).

## Supplementary Material

Supplementary Information

## Figures and Tables

**Table 1:** Elemental composition (C, N, H) of ATB and MBC.

Sample	N[%]	C[%]	H[%]
ATB	0.89	49.78	3.280
ATB	0.92	49.66	3.240
MBC	0.15	10.69	1.532
MBC	0.18	10.36	1.462

ATB: acid treated biochar.

MBC: magnetic Fe_3_O_4_ coated biochar.

**Table 2:** Adsorption capacities of reported adsorbents for crystal violet.

Adsorbent	Optical pH	*Q*_*e*_ (mg/g)	Reference
Jute fiber carbon	8.0	27.99	27
Coniferous pinus bark powder	8.0	32.78	28
TiO_2_-based nanosheet	8.5	58.30	29
sodium dodecylsulfate	7.0	76.90	30
Palm kernel fiber	8.0	78.90	31
carAlg/MMt Nanocomposite hydrogels	6.4	88.80	32
Opal	7.2	101.13	33
Cellulose	9.0	112	1
Magnetic nanocomposite	8.5	113.13	4
Cellolose-based adsorbent	9.0	182.15	1
Fe_3_O_4_ coated biochar	**6.0**	**349.40**	**This study**

**Table 3:** Parameters of pseudo-second-order kinetic model for the adsorption of crystal violet onto Fe_3_O_4_ coated biochar.

C_*0*_(mg/mL)	Q_*e,exp*_(mg/g)	Pseudo-second-order kinetic model		
		*K*_*2*_ × 10^−6^(g/mg·min)	*Q*_*e,cal*_	*R*^*2*^
50	49.75	1.02	49.11	0.9802
100	99.18	0.42	98.11	0.9810

**Table 4:** Parameters of Weber-Morris model for the adsorption of crystal violet onto Fe_3_O_4_ coated biochar.

Initial concentration(mg/L)	Initial linear portion		
	*K*_*w*_ × 10^−2^	*I*	*R*^*2*^
50	5.1529	48.9	0.9932
100	8.1612	97.79	0.9942

**Table 5:** Adsorption isotherm parameters for the adsorption of crystal violet onto Fe_3_O_4_ coated biochar at various temperatures.

*T*(°C)	*Q*_exp_(mg/g)	Langmuir constants			Freundlich constants			Temkin constants		
		*Q*_*max*_ (mg/g)	*K*_*L*_ (L/mg)	*R*^*2*^	*n*	*K*_*F*_ (L/mg)	*R*^*2*^	*A*_*T*_ (L/mol)	*B*_*T*_ (J/mol)	*R*^*2*^
20	279.43	278.55	0.071	0.984	3.65	69.43	0.977	0.26	59.23	0.946
30	292.14	289.91	0.089	0.981	4.07	83.51	0.971	0.24	894	0.980
40	349.49	356.71	0.870	0.999	4.00	171.80	0.978	0.24	1045	0.979

**Table 6:** Thermodynamic parameters for the adsorption of crystal violet onto Fe_3_O_4_ coated biochar.

*T* (°C)	*K*_*c*_	*ΔG*^*0*^ (KJ/mol)	*ΔH*^*0*^ (KJ/mol)	*ΔS*^*0*^ (J/mol·K)
20	2.24	−1.96	42.68	151.23
30	2.71	−2.51	—	—
40	6.90	−5.09	—	—
