# Supplementary material for: Efficient removal of crystal violet using Fe3O4-coated biochar: the role of the Fe3O4 nanoparticles and modeling study their adsorption behavior
Source: Sci Rep. 2015 Jul 29;5:12638. doi: 10.1038/srep12638 (PMC4518237; doi:10.1038/srep12638)
Supplement: Supplementary Information [file srep12638-s1.doc]

# Efficient removal of crystal violet using Fe_3_O_4_-coated biochar: the role of the Fe_3_O_4_ nanoparticles and modeling study their adsorption behavior

Pengfei Sun^a^, Cai Hui^a^, Rashid Azim Khan^a^, Jingting Du^b^, Qichun Zhang ^c^, Yu-Hua Zhao^a^*

*^a^* *College of Life Sciences, Zhejiang University, 310058 Hangzhou, Zhejiang, PR China*

*^b^ School of Forestry and Biotechnology, Zhejiang Agriculture and Forestry University, 311300 Lin’an, Zhejiang, PR China*

*^c^ College of Environmental and Resource Sciences, Zhejiang University, 310058 Hangzhou, Zhejiang, PR China*

* Corresponding author. Tel.:+86 571 88208557

E-mail address: yhzhao225@zju.edu.cn (Y.H. Zhao)


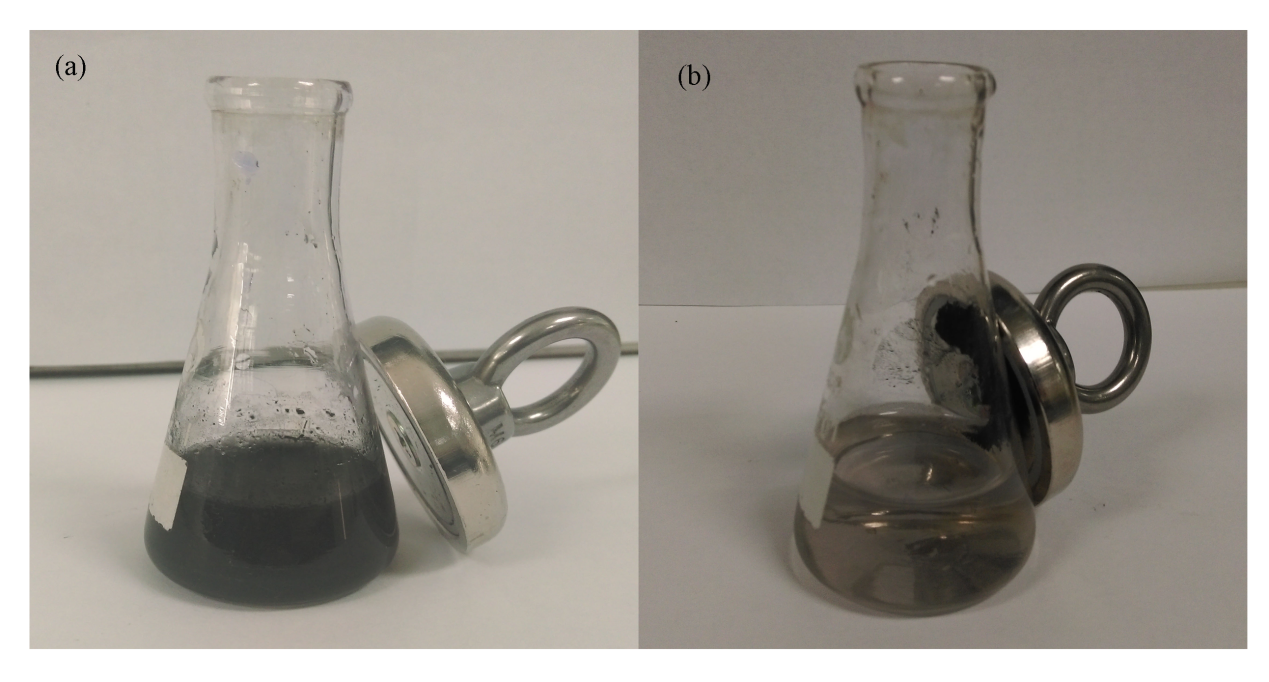


Figure S1 Re-collection of two different biochar by a magnet after their use in treating crystal violet solution. (a) The acid treated biochar; (b) The magnetic biochar.
